# Immunosuppressive Therapy in Immune-Mediated Liver Disease in the Non-Transplanted Patient

**DOI:** 10.3390/ph7010018

**Published:** 2013-12-30

**Authors:** Anita Abhyankar, Elliot Tapper, Alan Bonder

**Affiliations:** 1 Department of Medicine, St. Elizabeth’s Medical Center, Tufts University School of Medicine, 736 Cambridge Street, Brighton, MA 02135, USA; E-Mail: anita_abhy@hotmail.co.uk; 2 Liver Center, Beth Israel Deaconess Medical Center, Harvard Medical School, 110 Francis Street Suite 8E, Boston, MA 02115, USA; E-Mail: etapper@bidmc.harvard.edu

**Keywords:** autoimmune liver disease, autoimmune hepatitis, primary biliary cirrhosis, primary sclerosing cholangitis, overlap syndrome

## Abstract

Autoimmune liver disease management goals are primarily slowing disease progression and symptomatic treatment. There are few options for curative medical management other than transplant for a spectrum of autoimmune liver disease that encompasses autoimmune hepatitis, primary biliary cirrhosis, primary sclerosing cholangitis as well as their overlap syndromes. These diseases are managed primarily with immunosuppressive therapy. Herein, we review the current literature, detailing the promise and pitfalls of the recommended immunosuppressive therapy for these challenging diseases.

## 1. Introduction

Immunosuppressant therapy is a critical component of treatment in autoimmune mediated liver disease. Owing to the complex nature of immune mediated organ injury, immunosuppressive therapy has two principal effects, each resulting in specific benefits but also harms. Immunosuppressants modulate or abolish immune processes which have the effect of dampening autoimmunity but they also suppress normal immune function. The utility and power of these medications is predicated on the clinician’s ability to balance their benefits and harms. Herein, we review the benefits as well as the risks of standard pharmacotherapeutics of the following immune mediated liver diseases: autoimmune hepatitis (AIH), primary biliary cirrhosis (PBC), primary sclerosing cholangitis (PSC) as well as their overlap syndromes.

## 2. Autoimmune Hepatitis (AIH)

AIH is an idiopathic, chronic if fluctuating hepatitis characterized histologically by a mixed (lymphocytic and plasma cell) portal and interface inflammatory infiltrate, often in the setting of suggestive epidemiology (e.g., female predominant) and autoantibodies (e.g., ANA, anti-smooth muscle antibodies (anti-SMA), anti-liver-kidney microsomal (anti-LKM) antibody and anti-soluble liver antigen (anti-SLA) [1,2].

Immunosuppressive therapy greatly improves survival and frontline therapy combines corticosteroids and azathioprine with induction of remission by corticosteroids alone or in combination [2]. Monitoring of response thereafter includes serial aminotransferase and immunoglobulin measurement [2]. Treatment should be continued beyond normalization of transaminases since histology change lags behind. Additionally, a liver biopsy should be performed at the time of diagnosis for as many as 30% of patients have established cirrhosis at presentation [2]. Prednisone has long been the mainstay of corticosteroid therapy however there is increasing experience with budesonide in this setting, which, owing to its more favorable side effect profile, is often considered first line. Below we discuss current evidence behind the various immunosuppressant options for AIH.

Standard therapy for treatment-naïve patients includes prednisone in combination with azathioprine (Table 1). The duration of induction therapy is highly variable and driven by the time to resolution of hepatitis. Fewer than 40%–50% of patients experience complete biochemical remission in 6 months [3,4]. However, by 2 years of therapy, up to 75% of patients can expect remission. Patients should be started on prednisone 40 mg per day (though many groups choose doses ranging from 30–60 mg) during induction. This dose should be carefully tapered by 5mg on a weekly basis with serial evaluation of liver enzymes to ensure resolving hepatitis toward a maintenance dose of 10 mg. Prednisone, as is well known, can result in weight gain, hirsutism, acne and a variety of poorly tolerated side effects. Unfortunately, cosmetic side effects can occur in 80% after 2 years [5].

Budesonide is a corticosteroid with 90% first-pass hepatic metabolism. The reduction of systemic corticosteroid distribution sharply limits the intensity of steroid side effects. Budesonide was first studied in a large trial in 2010 using 6–9 mg daily in divided doses [3]. Combined with azathioprine for six months, budesonide resulted in complete biochemical remission in 60% of patients *versus* 38.8% with fewer side effects (28% *vs.* 53%) than prednisone combined with azathioprine. Notably, this trial excluded patients with cirrhosis, liver failure and co-morbid liver disease (including PBC and PSC). In general, corticosteroids are contraindicated in patients with fulminant liver failure, failing to improve outcomes while increasing infectious complications [6].

Corticosteroids are requisite first-line therapy for induction of remission. Thereafter, maintenance therapy is typically commenced with azathioprine. In general, it is successful in about 80% patients [7]. Johnson *et al*. [7] assigned 72 patients in remission for at least one year on low dose prednisone plus 1 mg/kg azathioprine to 2 mg/kg azathioprine alone. Sixty patients (83%) remained in remission for a median of 67 (12–128) months. Four patients had myelosuppression (multiple cell lines afflicted), 2 of which relapsed when the azathioprine was withdrawn. Many more developed lymphopenia. There is little international consensus on two important issues regarding this drug. First, in America, the common initial dose is 50–100 mg daily with prednisone during induction and 50–200 mg per day as monotherapy during maintenance. In Europe, a weight-based approach is favored with 1–2 mg/kg body weight depending on the phase of therapy. Second, there is no guideline consensus on whether to test for thiopurine methyltransferase enzyme activity to stratify for risk of toxic side effects. Clearly, however, the dose can be adjusted to optimize risks and benefits. One can also consider using 6-mercaptopurine (azathioprine is its pro-drug) or 6-thioguanine (the active metabolite of both), though the published experience is limited [8,9].

**Table 1:** Immunosuppressive Pharmacotherapy—Autoimmune Hepatitis.

Drug	First Line Therapy
Prednisone	First line therapy induction therapy. Remission in ≤ 50% within 6 months and 75% by 2 years. Combine with azathioprine. Start with 30–60 mg and taper toward 5–15 mg guided by liver enzymes. Has well-known toxicities: metabolic, cosmetic and psychiatric.
Budesonide	Potentially first line induction therapy (in selected patients without comorbid illness or cirrhosis). Reduced systemic distribution due to 90% first-pass metabolism. May be more effective than prednisone with 60% achieving biochemical remission in 6 months (compared to 38%).
Azathioprine	First line maintenance therapy. It is a steroid-sparing antimetabolite capable of maintaining remission in roughly 80% of patients. Myelosuppressive and hepatotoxic side effects require frequent blood tests after initiation.
	**Second line therapy**
Cyclosporine	Second line induction and maintenance therapy. Experience is derived from case-reports with widely variable doses employed. Outcome data is not robust. Calcineurin-inhibitor class-specific side effects including hypertension, nephrotoxicity and neurotoxicity.
Tacrolimus	Second line induction and maintenance therapy. Potentially the better tolerated calcineurin-inhibitor with even less robust supporting literature than cyclosporine.
Mycophenolate Mofetil	Second line maintenance therapy. An antimetabolite with very mixed results. Best used in patients intolerant of rather than those who have failed azathioprine.

Alternate regimens derivative from experience in other diseases are many, though supporting literature may be sparse. Cyclosporine, a calcineurin inhibitor, is a promising agent for the 10% of patients with steroid refractory disease or those who are known to be corticosteroid intolerant [10]. For patients failed by front-line corticosteroid therapy, a trial of cyclosporine at 2–5 mg/kg daily in divided doses can be considered with positive outcomes in as many as 93% of patients, albeit in case-report level data [11,12,13]. It is not clear from the literature whether a particular trough level ought to be targeted, though 100–250 ng/mL is suggested by some groups [11,14,15]. The class specific side-effects of calcineurin inhibitors are notable and include hypertension and drug-level dependent renal dysfunction and neurotoxicity. However, amongst the pediatric population, cyclosporine use in AIH has been shown to have better tolerance with milder side effects including hypertrichosis and gingival hypertrophy with similar dosing and outcomes compared to adults [16,17]. Similarly, tacrolimus, a second-generation calcineurin inhibitor, has also been used. In a case-series from van Thiel *et al*. [18], 21 patients followed for a year received tacrolimus at 3 mg twice daily with median trough levels of 0.5 ng/mL resulting in a 70%–80% reduction in aminotransferases.

For patients with contraindications to or intolerance of azathioprine, a different antimetabolite drug, Mycophenolate mofetil (MMF), has been investigated. The published experience with this drug is mixed and dates to 1998 and 2000 with the first reports by Schuppan and Richardson [19,20]. Inductivo-Yu [21] reported that, at typical doses (1 gram twice daily), 15 azathioprine intolerant patients, three on MMF monotherapy and 12 on prednisone and MMF, experienced favorable outcomes with reductions in biochemical as well as histological markers. The following year, Hlivko *et al*. [22] reported their series of 29 patients who received MMF, 12 of whom were switched to MMF after intolerance or nonresponse to prednisone-azathioprine and 17 of whom received MMF-prednisone as initial therapy. Their results present a characteristically mixed picture. Of the 29 patients who received MMF, 34% discontinued it as a result of side effects while 16 (84%) of the remaining patients on MMF achieved remission. It appears that failure to respond to azathioprine predicts failure of MMF. Of the eight patients treated by Hennes *et al*. [23] with MMF who had prior nonresponse to azathioprine, six (75%) did not respond to MMF. Meanwhile, of the 28 patients in their series with azathioprine intolerance, remission was reached in 12 (43%). MMF’s side effects include myelosuppression, notably leukopenia and gastrointestinal distress.

Finally, it is likely that ursodeoxycholic acid (UDCA) has immunomodulating properties. However, it is not particularly effective for AIH without an overlap syndrome. It can be used in combination with prednisone plus or minus azathioprine [2]. Czaja *et al*. [24] enrolled 37 subjects with refractory disease and randomized them to receive either placebo or 13–15 mg/kg of UDCA daily with corticosteroids. After 6 months of therapy, patients treated with UDCA were no more likely to have a dose reduction in corticosteroids or attain biochemical or histological improvement.

## 3. Primary Biliary Cirrhosis (PBC)

PBC is characterized by chronic bile duct inflammation resulting in cholestasis and liver injury manifesting most frequently as fatigue and pruritus with elevated serum alkaline phosphatase. Up to 90% of patients with PBC are women. The diagnosis of PBC is based on the presence of cholestasis, a positive antimitochondrial antibody (AMA) which is present in as much as 95% of cases or diagnostic liver biopsies [25]. Overlap syndromes have also been described between AIH-PBC.

Frontline therapy consists of UDCA for induction and maintenance therapy at a dose of 13–15 mg/kg divided into two daily doses (Table 2). UDCA is the only FDA approved drug for treatment of PBC. A systematic review of 16 treatment trials including 1,447 patients showed biochemical and symptomatic improvement with UDCA, but failed to show any mortality benefit [26]. In a combined analysis of three randomized, placebo controlled trials performed in France, America and Canada including 548 patients, UDCA treated was associated with a reduced likelihood of liver transplantation or death after up to 4 years of follow up [27]. UDCA has been shown to slow disease progression [27,28,29]. At ten years, treated patients have significantly better survival than non-treated patients and only slightly lower survival than age- and sex-matched controls from the general population [27]. In general, most investigators argue that early initiation and prolonged follow up gives the best results. Expected side effects of UDCA include headaches and gastrointestinal symptoms.

**Table 2:** Immunosuppressive Pharmacotherapy—Primary Biliary Cirrhosis.

Drug	First Line Therapy
Ursodeoxycholic Acid	First line induction and maintenance therapy. Combine with budesonide. If used as monotherapy start with 13–15 mg per kg per day.
	**Second Line Therapy**
Methotrexate	Second line induction and maintenance therapy. Use in UDCA refractory patients. Monotherapy 7.5 mg per week. Combine with colchicine. Hepatotoxic and myelosuppressive side effects require frequent blood tests.
Colchicine	No evidence for monotherapy. Combined therapy with UDCA results in improved biochemical parameters without improvement in outcomes.
Prednisone	No evidence of benefit.
Azathioprine	No evidence of benefit.
Cyclosporine	Controversial, limited evidence.

Several groups have looked at combination therapy with UDCA. Colchicine and methotrexate are the most common co-therapies. While early results were encouraging, methotrexate, generally given at 0.25 mg/kg weekly, has had mixed results over time. Bonis *et al*. [30] added low dose methotrexate to the treatment regimen (UDCA and colchicine) of 10 patients who were not responding to therapy with significant biochemical improvements. Kaplan *et al*. [31] compared patients with PBC who received UDCA plus methotrexate *versus* UDCA plus colchicine. No difference in survival was found and neither colchicine nor methotrexate improved transplant free survival above that predicted by a conventional risk assessment tool—Mayo prognostic model—which incorporates age, synthetic function (bilirubin, albumin and prothrombin time) and volume status (peripheral edema and diuretic use) [32]. Combes *et al*. [29] performed a randomized trial with 265 randomized to UDCA plus methotrexate and 133 to UDCA and placebo. In this study, methotrexate, where added to UDCA for a median of 7.6 years, showed no significant effect on disease progression or survival. The study was stopped early for futility.

Again, early data was supportive of colchicine. Colchicine, commonly at a dose of 1.2 mg daily in divided doses, has been shown most helpful in symptom control and can show biochemical improvement [31,33]. In a small trial of 90 patients randomly assigned to UDCA (at a suboptimal dose of 500 mg daily) with or without colchicine, Almasio *et al*. [34] showed that the addition of colchicine to UDCA resulted in a small but significant reduction of disease progression. However, as above, in the later trials, there was no benefit [31]. A systematic review studying 631 patients showed no improvement in mortality [35]. The balance of the evidence for colchicine so far has shown no improvement in disease progression [35,36,37,38,39].

In patients with UDCA-refractory PBC, additional therapy should be considered. Kaplan *et al*. [40] studied methotrexate and colchicine in UDCA refractory PBC. This study investigated 91 PBC patients who failed to respond to UDCA after 1 year of treatment were started on colchicine for 6 months and if there was no response then methotrexate (0.25 mg/kg per week) was added. Their results showed a significant improvement in biochemical tests and histology in 73 of 91 patients. Drugs such as prednisone, cyclosporine, azathioprine and d-penicillamine are generally not recommended owing to their toxic side effects and limited clinical experience [41,42,43,44].

## 4. Primary Sclerosing Cholangitis (PSC)

PSC is a chronic, progressive disease of the bile ducts. The pathogenesis is thought to be autoimmune. There is also strong association with inflammatory bowel disease. The long-term sequelae of PSC include cirrhosis and a 10%–20% lifetime risk of cholangiocarcinoma.

There is no FDA approved drug for PSC. This is consistent with 2010 AASLD guidelines which do not recommend UDCA for PSC treatment because of insufficient proven benefit on mortality or liver transplantation [45]. Many studies have investigated the benefit of UDCA. Earlier studies suggested that UDCA therapy (13–15 mg/kg) could attain biochemical improvement [46] (Table 3). However, by 1997, the results of high quality trials were less favorable when the Mayo PSC group published their randomized trial concluding that UDCA failed to improve outcomes even while it improved biochemical features [47]. Two large studies followed. First, Olssen *et al*. [48] reported their randomized controlled trial where 219 patients were randomized to either 17–23 mg/kg UDCA or placebo for 5 years. In this trial, the combined end point of death or liver transplantation occurred in 7.2% of patients receiving UDCA *versus* 10.9% of patients receiving placebo (*p* = 0.368). Lindor *et al*. [49] reported a multicenter, randomized placebo controlled trial in 2009 that was terminated early for futility. One hundred and fifty patients were enrolled and randomized to UDCA doses of 28–30 mg/kg or placebo. During therapy, biochemical tests improved in the UDCA group compared to placebo but these improvements were not associated with clinical benefit. The relative risk of a reaching primary endpoint was 2.3 times greater for patients on UDCA (30 *versus* 19 patients) and 2.1 times greater for death, transplantation, or minimal listing criteria (22 *vs.* 15 patients). Notably 15 patients on UDCA also developed esophageal or gastric varices compared to five on placebo.

There is insufficient evidence on the use of steroid, calcineurin inhibitors, methotrexate, azathioprine and penicillamine therapy.

**Table 3:** Immunosuppressive Pharmacotherapy—Primary Sclerosing Cholangitis.

Drug	No identified first line therapy
Ursodeoxycholic Acid	Most evidence available. Associated with higher risk of adverse events including the development of varices
Prednisone	Insufficient evidence
Azathioprine	Insufficient evidence
Methotrexate	Insufficient evidence

## 5. Overlap Syndromes

Autoimmune hepatitis, PBC and PSC may co-exist as a so-called overlap syndrome. Treatment is derivative of published experience with the primary diseases. Overlap syndromes are described anywhere between 5%–20% of AIH patients by clinicians [50]. Below we discuss treatment options for overlap syndromes.

### 5.1. AIH-PBC

The data on treatment and even diagnosis of AIH-PBC is limited. The longest standing criteria, “Paris criteria” suggest that patients with overlap have PBC defined by: (i) alkaline phosphatase levels at least twice the upper limit of normal, (ii) positive AMA, and (iii) a liver biopsy showing florid bile duct lesion and AIH defined by (i) alanine transaminase levels at least five times the upper limit of normal, (ii) Immunoglobulin G levels at least twice the upper limit of normal or a positive anti-SMA, and (iii) a suggestive liver biopsy [51]. Some reports suggest that it may be underdiagnosed or not detected when the disease transitions from AIH to overlap. Confirming earlier data, Bonder *et al*. [52] argued this point in a retrospective, single institution review. In this study, several patients who did not meet Paris Criteria had a complete response to prednisone, confirming a major component of AIH [52,53]. Therapy is, at this point, a hybrid of front-line therapy for both conditions. Chazouilleres *et al*. [54] looked at 17 AIH-PBC overlap patients. Eleven received UDCA alone and six received UDCA and corticosteroids. Twenty-seven percent in the UDCA alone group showed a biochemical and immunological response whereas 67% in the combination therapy group showed biochemical response. Combination treatment with UDCA (13–15 mg/kg) and corticosteroids (+/− azathioprine) is recommended based on non-randomized non-blinded studies [50].

### 5.2. AIH-PSC

A study by Floreani *et al*. [55] reviewed seven patients (7/41 included patients) followed for at least 2 years. Diagnosis of AIH-PSC was made by: a total AIH score of more than 15; ANA or anti SMA of at least 1:40 and based on histology. Their results showed biochemical improvement and survival benefit with combination induction therapy with UDCA 15–20 mg/kg, prednisolone 0.5 mg/kg and azathioprine 2 mg/kg, followed by maintenance daily dosing with prednisolone 10–15 mg and 50–75 mg azathioprine plus UDCA as above stated dose.

## 6. Conclusions

In this review we give an overview of the use of immunosuppressants in chronic liver disease, specifically AIH, PBC and PSC. Immunosuppressants have many different functions ranging from slowing disease progression to purely symptomatic control and it is often a balance between clinical improvement and toxic side effects. This is a dynamic field with questions yet to be answered about the exact mechanism of disease and discovery of new treatments.
